# Docetaxel and Lidocaine Co-Loaded (NLC-in-Hydrogel) Hybrid System Designed for the Treatment of Melanoma

**DOI:** 10.3390/pharmaceutics13101552

**Published:** 2021-09-24

**Authors:** Ludmilla David de Moura, Lígia N. M. Ribeiro, Fabíola V. de Carvalho, Gustavo H. Rodrigues da Silva, Priscila C. Lima Fernandes, Sérgio Q. Brunetto, Celso D. Ramos, Lício A. Velloso, Daniele R. de Araújo, Eneida de Paula

**Affiliations:** 1 Department of Biochemistry and Tissue Biology, Institute of Biology, University of Campinas—UNICAMP, Campinas 13083-862, SP, Brazil; ludmilladavidm@gmail.com (L.D.d.M.); nuneslica@gmail.com (L.N.M.R.); fabiolavieiracarvalho@hotmail.com (F.V.d.C.); gustavohrs@gmail.com (G.H.R.d.S.); priscila_biologa@yahoo.com.br (P.C.L.F.); 2 Institute of Biotechnology, Federal University of Uberlândia—UFU, Uberlândia 38405-319, MG, Brazil; 3 Radiology Department, University of Campinas—UNICAMP, Campinas 13083-887, SP, Brazil; brunetto@unicamp.br (S.Q.B.); cdramos@unicamp.br (C.D.R.); 4 Clinical Medicine Department, School of Medicine Science, University of Campinas—UNICAMP, Campinas 13083-887, SP, Brazil; lsincel@unicamp.br; 5 Human and Natural Science Center, ABC Federal University—UFABC, Santo André 09210-580, SP, Brazil; daniele.araujo@ufabc.edu.br

**Keywords:** nanostructured lipid carriers, hydrogel, lidocaine, docetaxel, melanoma

## Abstract

Melanoma is the most aggressive skin carcinoma and nanotechnology can bring new options for its pharmacological treatment. Nanostructured lipid carriers (NLC) are ideal drug-delivery carriers for hydrophobic drugs, such as the antineoplastic docetaxel (DTX), and hybrid (NLC-in-hydrogel) systems are suitable for topical application. This work describes a formulation of NLC_DTX_ in xanthan-chitosan hydrogel containing lidocaine (LDC) with anticancer and analgesia effects. The optimized nanoparticles encapsulated 96% DTX and rheological analysis revealed inherent viscoelastic properties of the hydrogel. In vitro assays over murine fibroblasts (NIH/3T3) and melanoma cells (B16-F10), human keratinocytes (HaCaT) and melanoma cells (SK-MEL-103) showed reduction of docetaxel cytotoxicity after encapsulation in NLC_DTX_ and HGel-NLC_DTX_. Addition of LDC to the hybrid system (HGel-NLC_DTX_-LDC) increased cell death in tumor and normal cells. In vivo tests on C57BL/6J mice with B16-F10-induced melanoma indicated that LDC, NLC_DTX_, HGel-NLC_DTX_-LDC and NLC_DTX_ + HGel-LDC significantly inhibited tumor growth while microPET/SPECT/CT data suggest better prognosis with the hybrid treatment. No adverse effects were observed in cell survival, weight/feed-consumption or serum biochemical markers (ALT, AST, creatinine, urea) of animals treated with NLC_DTX_ or the hybrid system. These results confirm the adjuvant antitumor effect of lidocaine and endorse HGel-NLC_DTX_-LDC as a promising formulation for the topical treatment of melanoma.

## 1. Introduction

Cutaneous melanoma is the most aggressive form of skin cancer due to the high rate (50%) of brain metastasis [1]. Although melanoma corresponds to only 2% of the registered cases of skin cancer, it is the cause of 80% of patient deaths [2]. The standard treatment of melanoma is the chemotherapy associated with surgical excision. However, melanoma’s high recurrence rate and low response and resistance to drug therapy [3,4] compel advances in treatments to improve patients’ survival. In this sense, nanotechnology developments can provide the sustained release of drugs, increasing their therapeutic efficiency and decreasing systemic toxicity [5].

Nanostructured lipid carriers (NLC) are drug delivery systems composed of a matrix of solid and liquid lipids stabilized by a surfactant [6,7]. In comparison to other lipid-based carriers such as liposomes and solid lipid nanoparticles, NLC have higher encapsulation efficiency and prolonged release times [8,9] for nonpolar drugs. Hydrophobic antineoplastic agents such as the semi-synthetic taxane docetaxel (DTX) are suitable candidates to be loaded in NLC. DTX is a cytostatic drug for the control of tumor tissue growth [10] widely used against breast, ovarian, prostate, non-small-cell lung cancer, melanoma, gastric adenocarcinoma and other cancer types [11]. DTX reversibly binds and promotes transitory microtubule stabilization, leading to cell cycle arrest [11].

Biopolymer-based hydrogels are also used as drug delivery carriers [12]. Alginate, xanthan, gelatin and chitosan are biocompatible, abundant, cheap [13,14,15] and bioadhesive biopolymers that promote fixation to the area of interest, allowing a more efficient drug release/biological activity in relation to ointments and creams [15,16]. Furthermore, hydrogels can be associated with nanoparticles to form nanohybrid systems [16,17,18] as shown in here for NLC_DTX_ in a xanthan-chitosan hydrogel containing lidocaine (HGel-NLC_DTX_-LDC).

Recently, several works have described the adjuvant anticancer action of local anesthetics such as LDC [19,20], bupivacaine [21,22,23,24] and ropivacaine [25]. LDC can suppress cancer cell growth (in vitro and in vivo) through several mechanisms: regulation of epigenetic changes, promotion of pro-apoptotic pathways and regulation of ABC transporters, also preventing metastasis and angiogenesis. However, these effects differ according to the cell line used and LDC shows time and dose-dependent cytotoxicity [26,27]. Therefore, a deep evaluation of the use of local anesthetics as anticancer agents is compelling, since their mechanisms of action may open new options for the cancer treatment [20,28,29].

In this work, a hybrid NLC-hydrogel is proposed as an innovative formulation for the treatment of melanoma. The hybrid hydrogel formulation uploads 2% (*w*/*v*) LDC incorporated in a (xanthan-chitosan) biopolymer matrix plus 0.5% (*w*/*v*) DTX incorporated in NLC. The formulation was structurally characterized, and in vitro and in vivo biological assays confirmed increased drug bioavailability, equivalent tumor regression and lower systemic toxicity than free DTX. The hybrid hydrogel carrying LDC and DTX is then proposed as an outstanding therapeutic alternative for the treatment of cutaneous melanoma.

## 2. Materials and Methods

### 2.1. Materials

Docetaxel powder (DTX) and lidocaine hydrochloride (LDC) were donated by Cristália Prod. Quim. Farm. Ltd.a (Itapira, SP, Brazil). Docetaxel trihydrate 20 mg mL^−1^ (DTX_T-HYD_) was kindly provided by Blau Pharmaceutica S.A. (Cotia, SP, Brazil). 18F-fluorodeoxyglucose (^18^F-[FDG]) was a gift from Cyclobras Ind. Com. Lab. Services Ltda (São Paulo, SP, Brazil). Pluronic F-68 (P68), Myristyl myristate (MM), Miglyol 812^®^ (MG), chitosan (CHT), xanthan (XAN), DMEM medium, fetal bovine serum and 3-(4,5-dimethylthiazol-2-yl)-2,5-diphenyltetrazolium bromide (MTT) were supplied by Sigma-Aldrich (St. Louis, MO, USA). Deionized water (18 MΩ) was obtained from an Elga USF Maxima ultra-pure water purifier. Phosphate-buffered saline (PBS), dimethyl sulfoxide and ultrapure water are from Laborclin (Pinhais, PR, Brazil), Vetec (São Paulo, SP, Brazil) and Barnstead™, Thermo Scientific (Thermo Scientific, Waltham, MA, USA), respectively. Murine fibroblasts (NIH-3T3) and murine melanoma (B16-F10) cells were purchased from American type culture collection (ATCC, Manassas, VA, USA). Human melanoma (SK-MEL-103) and human keratinocytes (HaCaT) cell line were purchased from Memorial Sloan Kettering cancer center (New York, NY, USA and CLS cell lines Service GmbH (Heidelberg, Germany), respectively. All other reagents were of analytical grade.

### 2.2. NLC Preparation

NLC formulations were prepared through the emulsification-ultrasonication method [30]. Briefly, an oily phase composed of MM (65% *w*/*w*), MG (35% *w*/*w*) and DTX (1% *w*/*w*) was heated to 55 °C. An aqueous phase consisting of a P68 solution (3% *w*/*w*) was heated to the same temperature and both phases were mixed under high-speed agitation (10,000 rpm) for 3 min in an Ultra-Turrax homogenizer (IKA Werke, Staufen, Germany). The mixture was then sonicated for 30 min in a Vibracell tip sonicator (Sonics & Mat. Inc., Danbury, CT, USA) at 60 W and 20 kHz, in alternating 30 s (on/off) cycles, to avoid sample overheating. After that, the samples were immediately cooled to room temperature in an ice bath. Control samples, with no DTX (NLC_CTRL_), were also prepared.

### 2.3. NLC Characterization

#### 2.3.1. Determination of Particle Size, Polydispersity and Zeta Potential

The particle size (hydrodynamic diameter) and polydispersity index (PDI) were measured by dynamic light scattering (DLS) while Zeta potentials (ZP) were determined by electrophoretic mobility, using a Nano ZS90 analyzer (Malvern Panalytical, Worcestershire, UK). For the determination of nanoparticles concentration Nanotracking analysis was used, in a NS300 (NanoSight, Amesbury, UK) equipment. The samples were diluted 1000× (DLS) or 5000× (NTA) in deionized water and measured in triplicate, at 25 °C. 

#### 2.3.2. DTX Quantification and Encapsulation in NLC

The quantification of DTX was done using high performance liquid chromatography (HPLC), in Varian ProStar (PS 325 UV-visible detector and PS 210 pump) equipment equipped with Galaxie Workstation software (Walnut Creek, CA, USA). Briefly, a Gemini^®^ 5 μm, C18 (Phenomenex^®^, Torrance, MD, USA) column, with a mobile phase composed of 0.1% *v*/*v* phosphoric acid:methanol (30:70 *v*/*v*) under a flow rate of 1 mL/min was used. Absorbance was followed at 210 nm and the injection volume was 10 μL. The limits of detection and quantification of the analytical method were 3.0 and 9.0 µg/mL, respectively. 

Firstly, NLC suspensions were prepared by the homogenization-ultrasonication method. Then, the total amount of DTX in the formulations was determined by diluting the samples in the mobile phase (*n* = 3) followed by centrifugation in order to disrupt the nanoparticles and provide phase separation. The encapsulation efficiency (%EE) of DTX in NLC formulations was determined by the ultrafiltration-centrifugation method [6]: 0.4 mL aliquots of the samples were transferred to a 10 kDa pore filtration unit (Millex, Milipore, Burlington, MA USA) coupled to Eppendorf tubes and centrifuged for 20 min at 4100 g; the filtered solution was collected and free DTX was quantified by HPLC. %EE was calculated, according to [31]:

(1) $$\%\mathrm{EE}=\frac{\;\mathrm{total}\;\mathrm{drug}\;-\;\mathrm{free}\;\mathrm{drug}\;}{\mathrm{total}\;\mathrm{drug}\;}\times 100$$

where total drug is the amount of DTX quantified in the NLC suspension and free drug represents the unloaded DTX fraction, determined in the filtrate. 

Drug loading, expressing the amount of DTX encapsulated per gram of nanoparticle [32] was also calculated:

(2) $$\%\;\mathrm{Drug}\;\mathrm{loading}=\frac{\mathrm{weight}\;\mathrm{of}\;\mathrm{encapsulated}\;\mathrm{drug}\;\left(g\right)}{\mathrm{weight}\;\mathrm{of}\;\mathrm{nanoparticles}\;\left(g\right)}\times 100$$

### 2.4. Hydrogel Preparation 

Chitosan was first solubilized in 0.1% acetic acid solution under constant magnetic stirring at 37 °C overnight, resulting in 3% chitosan solution (*w*/*v*). Xanthan hydrogel (5% *w*/*v*) was them prepared by dissolving the biopolymer in deionized water, under constant magnetic stirring, until complete homogenization. After that, biopolymer blends with different chitosan:xanthan mass ratios were prepared (Table S1). The selection of the most suitable biopolymer matrix considered the highest possible chitosan concentration, without inhomogeneities. A blend matrix containing 3% (*w*/*w*) XAN and 1% (*w*/*w*) CHT was chosen as the biopolymer component (XAN-CHT) of the hybrid hydrogel. 

For the hybrid hydrogel preparation, nanoparticle suspensions containing or not 1% DTX (NLC_DTX_ or NLC) were added to XAN-CHT matrix in a 1:1 (*w*/*v*) ratio and mixed under high-speed agitation (1200 rpm) for 30 s in an Ultra-Turrax homogenizer (IKA Werke, Staufen, Germany). Then, 2% LDC was dispersed in the XAN-CHT hydrogel samples and stirred for 2 h at room temperature. The prepared hydrogels (with final concentrations of 0.5% DTX and 2% LDC) were stabilized overnight at 4 °C and visually inspected before use. 

The same method was used to prepare all kind of hydrogels: control—without NLC or LDC (HGel_CTRL_); with NLC only (HGel-NLC); with LDC only (HGel-LDC); with NLC and DTX (HGel-NLC_DTX_) and with NLC_DTX_ plus LDC (HGel-NLC_DTX_-LDC).

### 2.5. Hydrogel Characterization

#### 2.5.1. Rheological Analyses 

Oscillatory rheometry measurements of control and hybrid hydrogels were performed in Kinexus Lab equipment (Malvern Instruments, UK) by using a cone plate geometry (20 mm diameter) in the range of 0.1–10.0 Hz, at constant shear stress (1 Pa) and temperature (32.5 °C, to simulate skin temperature application). Temperature scanning analyzes were also performed (from 10–50 °C) at a defined frequency (1 Hz), heating rate of 5 °C/min and 1 Pa shear stress. The storage module (G’), loss module (G”) and dynamic viscosity (η) were calculated using the Kinexus Rheometer rSpace software (NETZSCH Thermal Analysis, Wittelsbacherstraße, Germany).

#### 2.5.2. Field Emission Scanning (FE-SEM) and Cryo-Electron Microscopy (Cryo-EM)

To elucidate the morphological properties of the hydrogels a Quanta Feg 250 FE-SEM equipment (FEI Co., Hillsboro, OR, USA) was used. An aliquot of each hydrogel formulation was deposited on metal stubs previously mounted with a carbon ribbon. The sample was left to dry in a silica-containing desiccator for 24 h and covered with gold in a Bal-Tec SCD 050^®^ metallizer (Balzers Union AG, Balzer, Liechtenstein) for further image analysis. 

Cryo-electron microscopy (cryo-EM) allowed the structural analysis of the nanoparticles inside the hydrogel. For this, a 300 Mesh Holey Lacey Carbon grid was used, and the grids were submitted to a glow discharge procedure (Pelco EasiGlow discharge system, Ted Pella^®^, Redding, CA, USA) of 20 mA for 10 s, to make them more hydrophilic. Then, grids were inserted in a Mark IV Vitrobot^®^ (Thermo Scientific, Waltham, MA, USA) where they received 3 μL of sample, left to fix for 20 s. Subsequently, an automatic transfer (3 s) was performed to dry the excess sample with a negative transfer force (blot force = −5). Finally, the grid was rapidly plunged into liquid ethane and wrapped into a liquid nitrogen environment. Measurements were made in a Talos F200X (Thermo Scientific, Waltham, MA, USA) microscope at 200 kV. Cryo-EM images were measured in fresh samples (1 day) and after 8 months of storage at 4 °C to observe possible changes in the samples’ structure over time.

### 2.6. In Vitro Tests

#### 2.6.1. Cell Viability Assay

The experiments were carried using murine fibroblasts (NIH/3T3) and murine melanoma cells (B16-F10) as well as in human keratinocytes (HaCaT) and human melanoma (SK-MEL-103) cells. The cells were cultured in DMEM medium supplemented with 10% fetal bovine serum and 1% antibiotic (100 IU mL^–1^ of penicillin and 100 μg mL^–1^ of streptomycin sulfate) up to the logarithmic phase of growth, in 75 cm^2^ culture flasks at 37 °C in a humid atm with 5% CO_2_. Cytotoxicity was assessed using the MTT test [33] in 96-well plates at a concentration of 1 × 10^4^ (B16-F10 and SK-MEL-103) and 2 × 10^4^ (NIH/3T3 and HaCaT) cells per well. First, the cytotoxicity of the control (drug-free) samples NLC_CTRL_ and HGel_CTRL_ was investigated. For this, the cells were treated with increasing concentrations (10^3–^10^13^ particles/mL for NLC_CTRL_ and 0.01–6.25% *w*/*w* hydrogel for HGel_CTRL_) corresponding to those used in the formulations. The formulations (DTX_T-HYD_, NLC_DTX_, NLC_DTX_ + LDC, HGel-NLC_DTX_, HGel-NLC_DTX_-LDC) were tested at increasing DTX (from 0.02, 0.1, 0.5, 2 and 8 µmol/mL) and LDC concentrations (0.03, 0.1, 0.6, 2.5 and 10 mmol L^−1^), for 24 or 72 h. Cell viability was determined by the MTT method and the half-maximal inhibitory concentration (IC_50_) was calculated. Statistical analyses were conducted by Two-way ANOVA with post-hoc Bonferroni (* *p* < 0.05; ** *p*< 0.01; *** *p* < 0.001), with the GraphPad Prism version 6.04 for Windows (GraphPad Software, La Jolla, CA, USA).

#### 2.6.2. Analysis of Colony Formation (Clonogenic Assay)

The experiments were carried out with the two melanoma cell lines: B16-F10 and SK-MEL-103, from mice and human, respectively. The cells (5 × 10^5^/well) were seeded in 6-well plates; after 24 h they were treated with IC_50_ concentrations of either DTX_T-HYD_, LDC, NLC_DTX_, NLC_DTX_ + LDC, HGel-NLC_DTX_ or HGel-NLC_DTX_-LDC samples and incubated under cell growth conditions. After 24 h, the cells were seeded again at 1 × 10^3^ cells/plate in 6-well plates. Every three days, the culture medium was replaced with DMEM until the 14th day of incubation. After that, the cells were fixed with 1:1 (*v*:*v*) methanol:acetone solution, washed with PBS and stained with 0.5% (*w*/*v*) crystal violet for 30 min and analyzed using stereomicroscope.

### 2.7. In Vivo Tests

Female adult (12 weeks old) C57BL/6J mice were obtained from the Multidisciplinary Center for Biological Research (CEMIB-UNICAMP). A total of 50 animals were used in the experiments. The experimental protocol was approved by the UNICAMP Institutional Animal Care and Use Committee (Nb. 5345-1/2019—approved—30 August 2019).

#### 2.7.1. Anesthetic Efficacy: Tail-Flick Test

For the anesthetic efficacy test, three groups (5/cage) of animals were used to evaluate the effect of HGel-LDC, HGel-NLC and HGEL-NLC_DTX_-LDC formulations. The mice were maintained with free access to food and water. For the test they were placed in a restraint over an analgesimeter, with a portion of the tail (5 cm from the top) exposed to the heat of a projector lamp (55 ± 1 °C). A thirty-seconds cut-off time was adopted to avoid any thermal injury [34], and the baseline (normal response to the noxious stimulus) was registered. For the blockage of the caudal nerve, hydrogel samples (0.025 g) containing 2% LDC were applied on the back of the mice tail and occluded. The analysis started 30 min after hydrogel administration and the data were recorded every 30 min during the first 1 h, and every 60 min up to the end of the experiment (3 h). Data were expressed as percentage of maximum effect (% MPE), and the area under the curves was calculated. The statistical analyses were performed by Two-way ANOVA plus Tukey post hoc, *n* = 6 using the GraphPad Prism version 6.04 for Windows (GraphPad Software, La Jolla, CA, USA).

#### 2.7.2. Anticancer Tests in Melanoma Mice

B16-F10 cells (10^6^ cells per mouse) were subcutaneously injected into the right flank of the mice for tumor induction [35]. Tumors were allowed to grow for 8 days (to approximately 100 mm^3^) prior to the treatments with the hydrogel formulations.

The animals were then randomly divided into seven groups (five mice/group): group 1 = control without tumor (Naive); group 2 = tumor + saline (Positive control, intratumorally administered, IT); group 3 = tumor + DTX_T-HYD_ IT; group 4 = tumor + HGel-LDC, topically administered (TP); group 5 = tumor + HGel-NLC_DTX_ TP; group 6 = tumor + HGel-NLC_DTX_-LDC TP; group 7: tumor + NLC_DTX_ IT + HGel-LDC TP. While animals in the positive control group received only saline (0.025 mL, IT) in the tumor region, the other groups were treated either with 0.025 g of hydrogel samples or 0.025 mL of NLC_DTX_ or DTX_T-HYD_, as specified above. The antineoplastic dosage (10 mg/kg of DTX) was based on previous studies in the literature [11,36,37]. The chosen LDC concentration (2%) in HGel-LDC and HGel-NLC_DTX_-LDC also considered the anesthetic’s clinical doses [38,39]. Six sessions of each treatment were carried out, with a one-day interval between sessions. The animals were observed daily and possible signs of clinical impairment (lethargy, inability to walk, and loss of body weight) were assessed. The animals’ body mass and food intake were quantified weekly, using a PR224 digital analytical scale (OHAUS^®^, Barueri, SP, Brazil). The tumor sizes at specific time points (pre- and post-treatment) were measured with a PD 200 (Vonder^®^, Jundiaí, SP, Brazil) digital caliper and tumor volume (TM, mm^3^) was calculated according to [40]:

(3) $$\mathrm{Tumor}\;\mathrm{volume}=\frac{\mathrm{tumor}\;\mathrm{length}\;\left(\mathrm{mm}\right)\;\cdot \;\mathrm{tumor}\;\mathrm{width}\;{\left(\mathrm{mm}\right)}^{2}}{2}$$

Tumor volume after 7 days of treatment was also determined from micro-PET/CT, as described further (Section 2.7.4.). The survival probability of the groups was calculated by the Kaplan-Meier method [41]. The non-parametric log-rank test was used to compare the curves between the groups. The GraphPad Prism version 6.04 (GraphPad Software, La Jolla, CA, USA) generated the statistical data, *p* < 0.05 was considered significant.

#### 2.7.3. Tumor Regression Analysis by In Vivo Imaging (Micro-PET/CT)

Micro-PET/CT analyzes were performed according to the protocol suggested by [42]. Post-treatment tumor regression was analyzed anatomically by computed microtomography (micro-CT), and metabolically by positron emission microtomography (micro-PET) [43]. The images were acquired in a micro-PET/SPECT/CT equipment (Bruker Biospin Corporation, Woodbridge, CT, USA) at the Preclinical Images Lab., School of Medical Sciences, UNICAMP. Tumor characterization was followed 7 days after the last treatment session. For the analyses, the animals were anesthetized with isoflurane (1–2%), placed in the animal carrier of the equipment, and fixed in the prone position to prevent movements during the scanning. Whole-body micro-CT of the animals was performed. To perform the micro-PET analyses, animals were injected with ~250 µCi of the radiopharmaceutical ^18^F-[FDG]. Micro-CT acquisition/reconstruction parameters were: total time 60 min, FOV 70 mm, 2 beds, kVp = 35, mA = 400 μA, projections = 1000, FBP and dering = 4. Micro-PET acquisition parameters were: total time 20 min, FOV 148 mm, 1 bed, MLEM = 6 interaction. Volumes of interest (VOIs) were drawn by the threshold technique and after logical/morphological operations the parameters%ID_MAX_ and%ID_Mean_ were obtained, for tumor (T), adjacent normal tissue (BG) and liver (L). From them, the ratios (%ID^[T/BG]^_Max_,%ID^[T/BG]^_Mean_,%ID^[T/L]^_Max_ and%ID^[T/L]^_Mean_) were calculated. Other semiquantitative metabolic parameters such as metabolic tumor volume (MTV) and total lesion glycolysis (TLG) were calculated [43].

#### 2.7.4. Serum Biochemical Analytes Measurement

Ten days after the last treatment session, the animals were euthanized by the intraperitoneal administration of ketamine (300 mg/kg) and xylazine (30 mg/kg) strictly following the guidelines of the National Committee of Animal Experimentation (CONCEA, Brasília, DF, Brazil). Immediately after euthanasia, blood samples were collected by cardiac puncture for biochemical analyzes (serum). Immediately after euthanasia, blood samples were collected from the animals by cardiac puncture (with 23 gauge needle and 1 mL syringe) and the biochemical analytes creatinine, urea (mg/dL), AST and ALT (IU/L) were measured in serum. The analyses were performed using appropriate kinetic methods, in an Hemovet 2300 automated device (São Paulo, SP, Brazil). 

#### 2.7.5. Histological Analysis 

After euthanasia and blood collection, the mice were placed on an operating field for removal of the lungs, liver, spleen, kidneys, and tumor/skin. All organs were weighed using an analytical digital scale. Histopathology of tumor, liver, lung, spleen, and kidney sections was evaluated using the hematoxylin and eosin (H&E) method. Tissues were fixed with paraformaldehyde, dehydrated, sectioned, and processed for H&E staining. Histopathological analyses were performed in an inverted Eclipse TS100 microscope (Nikon Instruments Inc., USA) with a color CMOS digital capture camera (Micron Scientific, Brazil) at 100× and 400× magnifications.

## 3. Results and Discussion

### 3.1. NLC_DTX_ Preparation and Characterization

The first challenge to prepare a DTX formulation is imposed by its low aqueous solubility. Thus, considering our previous experience in the encapsulation of hydrophobic drugs [44,45,46] we decided to encapsulate DTX into NLC. For that, a 1% (*w*/*w*) NLC_DTX_ formulation and its control (without drug, NLC_CTRL_) were prepared. As shown in Table 1, the incorporation of DTX did not significantly change the average size, polydispersity and zeta potential of the NLC. The physical stability of the nanoparticles, as predicted by the homogeneity of their size distribution (PDI < 0.15) and ZP values different from zero, was kept for 12 months of storage at 25 °C (Figure S1, Supplementary Material). As expected [11], the high encapsulation efficiency (>97%) and drug load capacity (7.5%) in Table 1 confirm the preference of DTX for the lipid environment of the nanoparticles.

### 3.2. Hybrid Hydrogel Development and Characterization

In the next step NLC were incorporated into hydrogels. For the development of XAN-CHT hydrogels (HGel_CTRL_), five blends with different proportions of each biopolymer (samples 1–5 in Table S1) were tested, to find the best consistency and homogeneity. The choice also considered the highest possible concentration of chitosan, to take advantage of its intrinsic antifungal, bactericidal and healing properties [47,48]. All blends but sample 1 showed suitable compatibility between the matrices. Sample 1 (Figure S2A), containing 2.5% xanthan + 1.5% chitosan, showed a heterogeneous aspect and was discarded. Sample 2, with 3.3% xanthan + 1% chitosan, was selected. LDC was then dispersed into this hydrogel, that remained translucent even after adsorption of the anesthetic (Figure S2B). Further incorporation of NLC turn the hydrogels whitish (as shown in Figure S2C,D).

**Figure 1:**
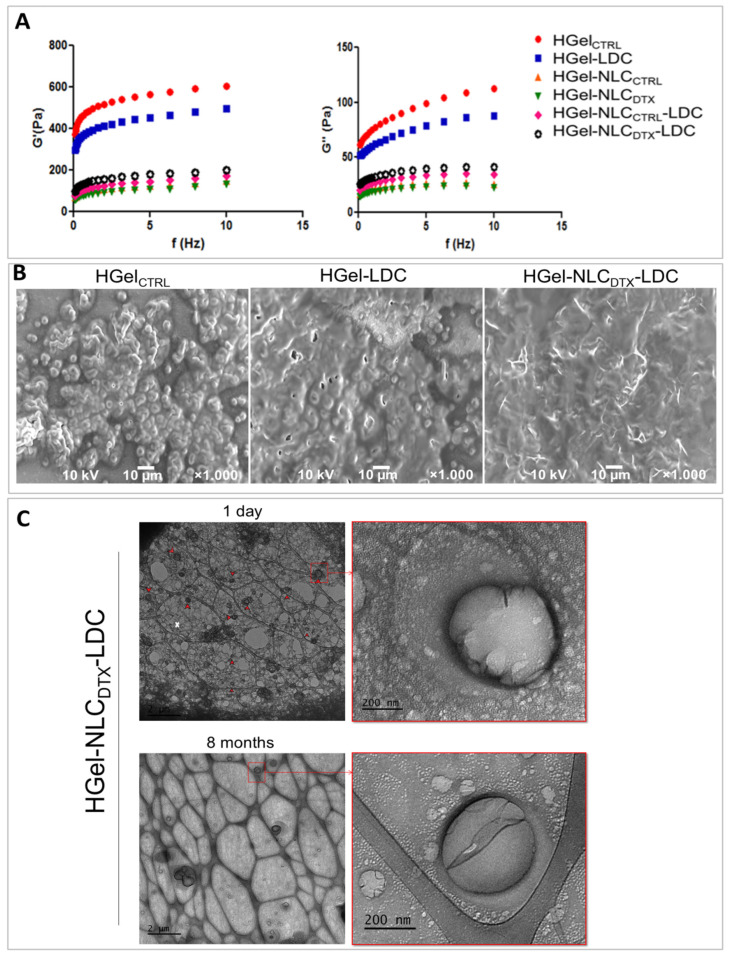
(**A**) Determination of the viscoelastic properties of hydrogels using oscillatory rheometry, at 32.5 °C: Elasticity (G’)-left-and viscosity (G”) modulus-right; (**B**) FE-SEM micrographs of the hydrogels: control (HGel_CTRL_), with lidocaine (HGel-LDC) and with lidocaine plus NLC_DTX_ (HGel-NLC_DTX_-LDC). Magnification: 1000×, 10 kV. (**C**) Cryo-EM of the hybrid hydrogel (HGel-NLC_DTX_-LDC), at 120 kV. Red arrows point to NLC_DTX_ dispersed in the freshly prepared hydrogel (**upper left**). Notice that the spherical morphology of the NLC was preserved when adsorbed in the hydrogel lattice, even after 8 months (**bottom**). Magnification: 60,000× (**left**) and 120,000× (**right**).

Oscillatory rheology measurements were then conducted with the different hydrogel samples: HGel_CTRL_ (control), HGel-LDC (with lidocaine), HGel-NLC_CTRL_ (with NLC-control), HGel-NLC_DTX_ (with NLC-docetaxel), and HGel-NLC_DTX_–LDC (with nanoparticles-docetaxel and lidocaine). The results in Figure 1A and Table S2 show the rheological parameters (G’, G”, G’/G” and ƞ) measured at 32.5 °C, the surface temperature of the skin. The frequency sweep analysis revealed the influence of nanoparticles and drug incorporation into the hydrogels structural network. Incorporation of both drugs reduced the apparent viscosity values and the G’/G” ratios when compared to control gels but maintained the prevalence of G’ over G” modulus. These results strongly suggest that all samples could be topically applied and recover their structure even under shear stress conditions such as those caused by spread on skin surface, improving the drug residence time on the application site [49].

On the other hand, the presence of the nanoparticles in the hybrid hydrogels (HGel-NLC_CTRL_ and HGel-NLC_DTX_) was accompanied by reduced G’/G” and apparent viscosity (ƞ) values (Table S2), suggesting NLC interference in the biopolymer network arrangement and hydrogel structural organization. 

It is worth mentioning that the pseudoplastic behavior was kept in all hybrid hydrogels tested at the skin temperature [13]. Such results indicate that the prepared hydrogels can be efficiently spread and preserve their structure without phase separation, being suitable for the application of drugs on the skin [49,50]. The polyelectrolyte complexes of XAN (anionic) and CHT (cationic) molecules are responsible for the tridimensional network stability and intrinsic therapeutic properties of this polymeric blend [51].

Morphological hydrogel characterization often uses scanning electron microscopy [52]. Here, FE-SEM micrographs were used to compare the morphological properties of HGel_CTRL_ and HGel-LDC and HGel-NLC_DTX_-LDC hydrogels. As shown in Figure 1B, control (HGel_CTRL_) and lidocaine-containing hydrogels (HGel-LDC) had amorphous appearance with wrinkled surfaces. Addition of NLC_DTX_ did not change the amorphous structure of the hydrogel, but apparently turned its surface more homogeneous, with a different texture than the control (HGel-NLC_DTX_-LDC sample, Figure 1B). 

To verify the morphology of NLC into the hybrid hydrogel, cryo-EM analysis was used. As expected, NLC_CTRL_ and NLC_DTX_ samples (suspensions) showed spherical and ellipsoidal structures [53,54], as shown in Figure S3. Most importantly, Figure 1C shows that in the hybrid sample, nanoparticles are distributed in the biopolymer matrix of the XAN-CHT hydrogel in which their spherical morphology and particle size were preserved, even after 8 months of storage (Figure 1C, middle/right).

### 3.3. In Vitro Tests

#### 3.3.1. Cell Viability Assay

**Figure 2:**
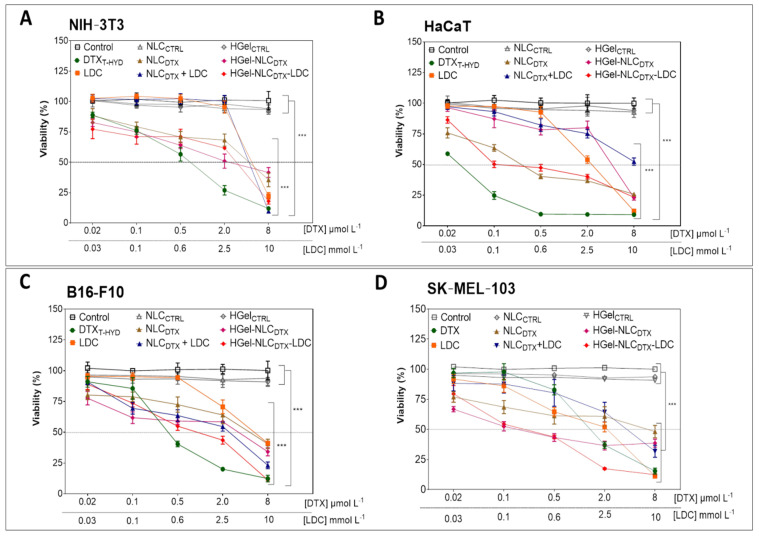
Viability of NIH-3T3 (**A**), HaCaT (**B**), B16-F10 (**C**) and SK-MEL-103 (**D**) cells after 24 h of treatment with DTX_T-HYD_, LDC, NLC_DTX_, NLC_DTX_ + LDC, HGel-NLC_DTX_ or HGel-NLC_DTX_-LDC at 0.02, 0.1, 0.5, 2 and 8 µmol L^−1^ (equivalent DTX concentrations) and 0.03, 0.1, 0.6, 2.5 and 10 mmol L^−1^(equivalent LD concentrations, evaluated by MTT assay. In the statistical analyses (by two-Way ANOVA plus Bonferroni post-hoc) each treatment was compared to untreated (control) cells. In NIH-3T3 and B16-F10 cells, the DTX_T-HYD_, NLC_DTX_, NLC_DTX_ + LDC, HGel-NLC_DTX_ and HGel-NLC_DTX_-LDC formulations showed a significant reduction (*** *p* < 0.001) in cell viability at the concentrations of 0.02 µmol L^−1^ docetaxel and above. The same pattern of cytotoxicity was observed in SK-MEL-103 cells, except for the DTX formulation for which a significant reduction in cell viability (*** *p* < 0.001) was only observed from the concentration of 0.5 µmol L^−1^ docetaxel on. For HaCaT cells the DTX, NLC_DTX_ and HGel-NLC_DTX_-LDC formulations promoted significant reduction (*** *p* < 0.001) in cell viability beginning at 0.02 µmol L^−1^ docetaxel, while the NLC_DTX_ + LDC formulations and HGel-NLC_DTX_ significantly reduced (*** *p* < 0.001) cell viability from the concentration of 0.1 µmol L^−1^ docetaxel and higher. Data expressed as mean ± SD (*n* = 3).

Four different cell lineages had their viability evaluated: melanoma cancer cells (B16-F10 and SK-MEL-103) and non-cancerous cells (NIH/3T3 and HaCaT). The results were compared with those induced by the commercial DTX formulation (docetaxel trihydrate—DTX_T-HYD_) and the survival of cells treated with control formulations (NLC_CTRL_ and HGel_CTRL_) was also investigated, to exclude any non-specific effects. Cells were treated at equivalent DTX (0.02, 0.1, 0.5, 2 and 8 µmol mL^−1^) and LDC (0.03, 0.1, 0.6, 2.5 and 10 mmol L^−1^) concentrations for 24 h (Figure 2) and 72 h (Figure S4). As expected, the control formulations (NLC_CTRL_ and HGel_CTRL_) were not toxic to any cell line, showing they are safe and suitable for use in intracellular applications. 

As for the proposed formulations, the results revealed a reduction in cell viability on non-tumor cells (NIH/3T3 and HaCaT, Figure 2A,B) and tumor cells (B16-F10 and SK-MEL-103, Figure 2C,D), treated with free (DTX_T-HYD_) or encapsulated docetaxel (NLC_DTX_, NLC_DTX_ + LDC, HGel-NLC_DTX_ or HGel-NLC_DTX_-LDC). Such effects were even more evident after 72 h, as shown in Figure S4. Table 2 shows the calculated half inhibitory concentration (IC_50_) determined after 24 h of treatment, for docetaxel and lidocaine. The IC_50_ value for unencapsulated drug DTX (DTX_T-HYD_) lied in the expected range of values (0.01–0.40 μmol/L) reported by previous studies in other tumor and non-tumoral cell lines [55,56]. 

Encapsulation into NLC_DTX_ and HGel-NLC_DTX_ determined significant (*p* < 0.05) reduction in the toxicity of DTX against all evaluated cell lines, within 24 h of treatment, as shown by the increased IC_50_ values (Table 2). Similar changes in cytotoxic response were observed by other authors that used lipid nanocarriers for DTX delivery [11,57] or a thermoreversible gel containing adsorbed NLC_DTX_ for the treatment of breast cancer [58]. In all cases, the authors attributed the decrease in cytotoxicity (regarding DTX_T-HYD_) to the sustained release of docetaxel from such hybrid systems, which also explains the increase in cytotoxicity registered after 72 h of treatment (Figure S4). 

On the other hand, free LDC had different dose-dependent cytotoxic profiles for each cell line (Figure 2). After 24 h the greater cytotoxic effect was observed with SK-MEL-103 cells, with reduction in cell viability from the concentration of 0.03 mmol L^−1^ on. For the HaCaT and B16-F10 strains, cytotoxicity was observed at concentrations higher than 0.6 mmol.L^−1^ and in NIH-3T3 cells a reduction in cell viability at 2.5 mmol.L^−1^ or higher LDC concentrations. Curiously, the IC_50_ of LDC (Table 2) was higher in murine (NIH-3T3: 4.95 ± 5.60 mmol L^−1^; B16-F10: 6.13 ± 5.44 mmol L^−1^) than in human (HaCaT: 2.67 ± 2.92 mmol L^−1^; SK-MEL-103: 3.26 ± 1.84 mmol L^−1^) lineages. Table 2 also shows that the addition of LDC to the NLC_DTX_ system (NLC_DTX_ + LDC) increased the cytotoxicity of the formulation against all cell lineages, as seen by the lower IC_50_ compared to NLC_DTX_ formulation. A closer look at Figure 2 in the highest tested concentrations (8 µmol L^−1^ DTX and 10 mmol L^−1^ LDC) demonstrates the potentializing cytotoxic effect of LDC on NIH-3T3, B16-F10 and SK-MEL-103 cells. The viability of NIH-3T3 cells (35% when treated with NLC_DTX_) decreased to 9% when LDC was added to the system (NLC_DTX_ + LDC); the same was observed for the hydrogel formulations: 52% cell viability with HGel-NLC_DTX_ and 18% with HGel-NLC_DTX_-LDC. For the B16-F10 tumor cells, viability was 40% when using NLC_DTX_ and 23% with NLC_DTX_ + LDC; in the hydrogels the viability decreased from 34% (HGel-NLC_DTX_) to 11% (HGel-NLC_DTX_-LDC). In the SK-MEL-103 tumor lineage cell viability (48% with NLC_DTX_) decreased to 32% with NLC_DTX_ + LDC; in the hydrogels the viability was 38% (HGel-NLC_DTX_) and 11% (HGel-NLC_DTX_-LDC). Only the HaCaT strain showed a different profile, with 26% cell viability after treatment with the NLC_DTX_ formulation and 52% when lidocaine was present (NLC_DTX_ + LDC) and almost no variation between the hydrogel formulations (23% for HGel-NLC_DTX_ and 24% for HGel-NLC_DTX_-LDC).

As for the cytotoxic effect of LDC, Karniel & Beitner demonstrated a significant and dose-dependent reduction in the activity of glucose 1,6-biphosphate and fructose 1,6-biphosphate in melanoma cells (B16) after treatment with the anesthetic, showing that its cytotoxicity was correlated with ATP depletion [59]. In 2008, Desai and coworkers verified that LDC affected the multiplication of NIH-3T3 cells, decreasing DNA synthesis by favoring the expression of cyclin 1A-dependent kinase inhibitor p21 [60]. Furthermore, in some resistant neoplastic cells such as B16-F10, LDC was found to act as a chemosensitizer to other conventional chemotherapeutics [26].

Overall, a time and dose-dependent cytotoxic activity was observed for all formulations and cell lines evaluated. Encapsulation decreased the toxicity of DTX while LDC intrinsic cytotoxicity explains the lower cell survivor rates observed in formulations containing DTX and the anesthetic (NLC_DTX_ + LDC vs. NLC_DTX_ and HGel-NLC_DTX_ + LDC vs. HGel-NLC_DTX_), against all cell lineages. 

#### 3.3.2. Evaluation of Cell Reproductive Viability (Clonogenic Assay)

**Figure 3:**
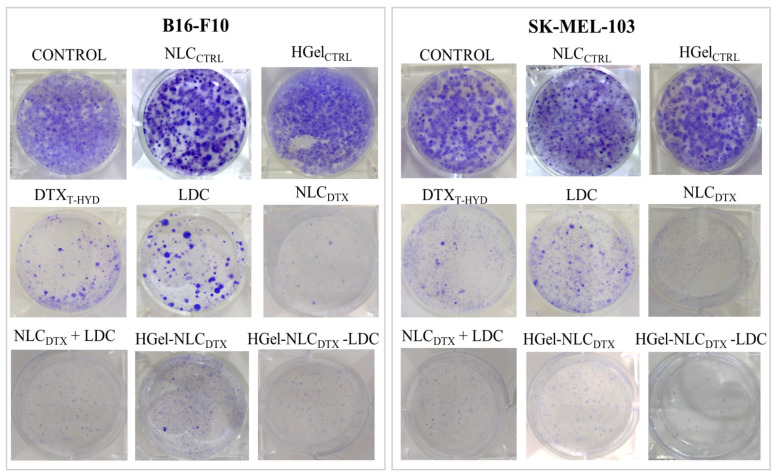
Clonogenic assay. Effect of the treatment with formulations on the ability of cancer (B16-F10) and SK-MEL-103) cells to form colonies.

The clonogenic assay is a consistent method to determine if the cell can proliferate/form colonies after being exposed to anti-tumor agents [61]. The top lane of Figure 3 shows that control (Naive) cells and those treated with NLC_CTRL_ and HGelC_CTRL_ were able to fully proliferate and form colonies, as a clear sign of no toxicity. On the other hand, the cancer cells treated with IC_50_ concentrations of free (DTX_T-HYD_ or free LDC, Figure 3—middle lane)—or encapsulated drugs (NLC_DTX_, NLC_DTX_ + LDC, HGel-NLC_DTX_ or HGel-NLC_DTX_-LDC, Figure 3—bottom lane) visually showed smaller number of colonies than non-treated cells. Such effect was evident for cells treated with either NLC_DTX_, NLC_DTX_ + LDC or HGel-NLC_DTX_-LDC formulations. The antiproliferative activity of LDC is also noticeable in Figure 3, against both cancer cell types. Such effect was less pronounced than that of free DTX_T-HYD_ or the formulations (see the IC_50_ values in Table 2). The clonogenic test agrees with the previously shown cell viability data (Table 2, Figure 2 and Figure S4).

Interestingly, more cell colonies were observed in cells treated with DTX_T-HYD_ in comparison to those treated with DTX incorporated into the NLC which should indicate that the cancer cells were less resistant to the cytotoxic effects of nanoformulations containing the antineoplastic than to free docetaxel. This behavior should be related to the internalization and sustained release of DTX promoted by the nanoparticles, since even the fraction of resistant cells (those that would be able to proliferate after treatment with DTX_T-HYD_) died after 24 h of treatment with the docetaxel-containing nanoparticles. Similar results were observed with other antineoplastic-based nanoformulations that promoted sustained release [62,63].

### 3.4. In Vivo Assays

#### 3.4.1. Anesthetic Efficacy Determined by the Tail-Flick Test

The tail-flick test is a commonly used method to determine the efficacy of local anesthetics in topical formulations [64]. This assay was performed to evaluate the anesthetic effect of LDC incorporated in the hydrogel formulations (Figure S5). The results of maximum possible effect (MPE) area under the curve (AUC) and anesthesia duration after topical application of hydrogels on the animal tail are shown in Table 3. 

All hydrogel formulations containing LDC showed similar analgesic effect: MPE after 30 min (Figure S5) and ca. 150 min of total anesthesia duration. No significant differences (* *p* < 0.05, Tukey’s multiple comparisons test) were observed among the formulations regarding MPE and AUC confirming that the antinociceptive effect of LDC was preserved in the hydrogels. These results corroborate previous reports in the literature, obtained with commercial LDC (2%) incorporated or not in gels and tested in mouse through the tail-flick test [65]. Therefore, the XAN-CHT hydrogel did not interfere with the anesthetic activity and could possibly contribute to the pharmaceutical formulation with the intrinsic anti-inflammatory, antifungal and bactericidal properties of the polymers [47,66,67]. 

#### 3.4.2. In Vivo Antitumor Efficacy in a Melanoma Model

**Figure 4:**
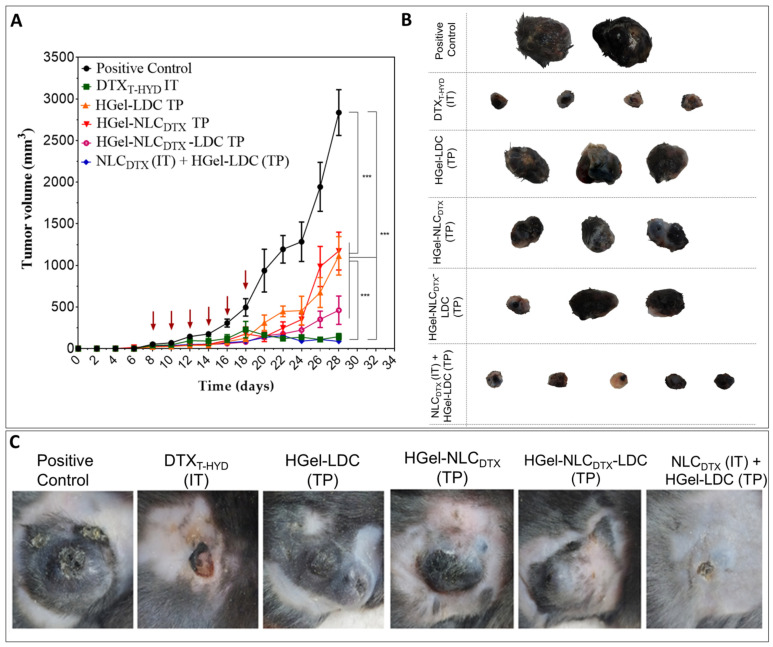
Antitumoral effect of saline (positive control), commercial Docetaxel (DTX_T-HYD_ IT), HGel-LDC TP, HGel-NLC_DTX_ TP, HGel-NLC_DTX_-LDC TP and NLC_DTX_ IT + HGel-LDC TP on B16-F10 tumor-bearing C57BL/6J mice. (**A**) Variation of tumor volume as a function of time after treatment (started at day 8th and subsequently applied on the 10th, 12th, 14th, 16th and 18th day, as indicated by the red arrows). (**B**) Photographs of tumors from each treatment before euthanasia. (**C**) Representative images of tumors excised from each treatment group. TP = topical application; IT = intratumor application. Statistical analysis: Two-Way ANOVA post-hoc Bonferroni (*** *p*< 0.001). Data expressed as mean ± SD (*n* = 5).

The in vivo therapeutic performance of nanohybrid hydrogels was studied in B16-F10 tumor-bearing C57BL/6J mice, treated with six doses of 10 mg/kg DTX. The dose was selected based on the previous works [11,36,37]. The tumor volume grew rapidly in mice treated with saline (positive control) than in those treated intratumorally (IT) or topically (TP) with conventional docetaxel formulation (DTX_T-HYD_ IT) or hydrogels (HGel-LDC TP, HGel-NLC_DTX_ TP, HGel-NLC_DTX_-LDC TP and NLC_DTX_ IT + HGel-LDC TP) as shown in Figure 4A. In comparison to the positive control all treated groups significantly reduced tumor growth (Two-Way ANOVA post-hoc Bonferroni, *** *p*< 0.001) (Figure 4B,C). 

The treatment with free DTX (DTX_T-HYD_ IT) inhibited tumor growth by about 95.0% at the end point of study. Treatments using the hydrogel with only LDC (HGel-LDC TP) or only NLC_DTX_ (HGel-NLC_DTX_ TP) inhibited tumor growth by 58.7% and 62.7%, respectively, while the inhibition with HGel-NLC_DTX_-LDC TP reached 75.4%. To compare the efficiency of the formulations with that of free DTX (DTX_T-HYD_), the group treated with NLC_DTX_ was also tested via intratumor administration associated to the topical application of lidocaine hydrogel (NLC_DTX_ IT + HGel-LDC TP). In this group, tumor growth inhibition reached 97.0% at the end of the experiments suggesting a possible synergistic effect between both therapeutic approaches and administration routes. 

The significant (*p* < 0.05) inhibition of tumor growth by HGel-LDC TP, HGel-NLC_DTX_ TP, HGel-NLC_DTX_-LDC TP and NLC_DTX_ IT +HGel-LDC TP discloses the effectiveness of the treatment using DTX-loaded NLC associated with LDC-based hydrogel. In addition, they reveal the potential of LDC for the treatment of melanoma, in association with an antineoplastic agent. Previous studies pointed out the anticarcinogenic effect of LDC in different tumor models, such as breast [68], bladder [20], squamous and basal cell carcinoma [69], gastric cancer [28] and melanoma cell [19]. A recent review shows that LDC acts as a chemosensitizer to other antineoplastic agents, inhibiting the growth of tumors after single use at different concentrations through the regulation of epigenetic modifications, promotion of pro-apoptotic pathways and angiogenic inhibitor. Its authors advise the repositioning of LDC as an antineoplastic agent [26]. Finally, in addition to the antiproliferative effect on tumor cells, LDC reduces pain and, consequently, may increase the patient compliance to the treatment [48,70]. 

Here, in the maximum antitumor therapeutic efficacy (equivalent to the conventional formulation with DTX_T-HYD_ IT) was observed when NLC_DTX_ was intratumorally administered together with the topical application of the LDC-based hydrogel. Such joint of administration routes has been previously proposed by other authors [36,71] and the increased therapeutic effect compared with the topical treatment could be attributed to the higher drug uptake at the tumor site [72,73,74]. These data open new possibilities for the future investigations of this hybrid system co-delivering LDC and DTX for the treatment of skin cancer.

#### 3.4.3. Tumor Regression Analysis by In Vivo Imaging

**Figure 5:**
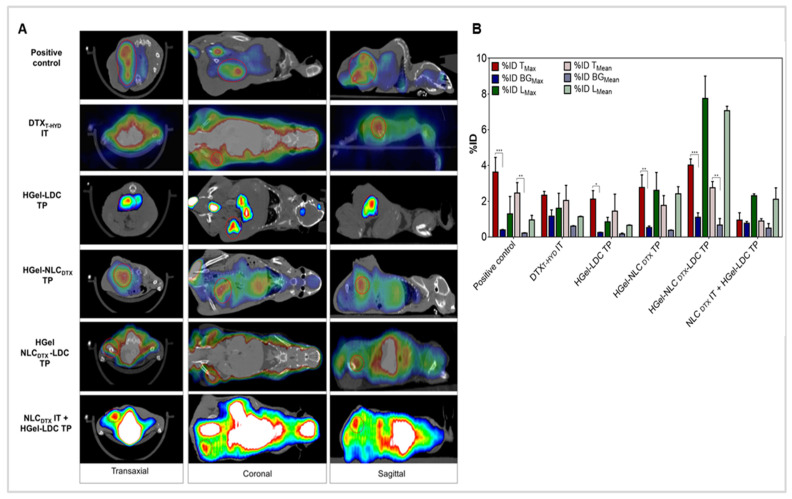
Micro-PET/CT imaging showing ^18^F-[FDG] uptake in mice with B16-F10 tumors treated with saline (Positive control), free docetaxel (DTX_T-HYD)_ and hydrogel formulations (HGel-LDC, HGel-NLC_DTX_ TP, HGel-NLC_DTX_-LDC TP and NLC_DTX_ IT + HGel-LDC TP). (**A**) Images taken 7 days after the end of the treatments. Additional identifiable structures with respect to ^18^F-[FDG] uptake were bladder, kidney, liver and heart. Dashed circles indicate the location of the tumor. (**B**) Quantification of ^18^F-[FDG] uptake (%ID_Max_ and ID%_Mean_, mean ± SD, *n* =2) in tumors (T), normal adjacent tissue (BG) and liver (L). The%ID_Max_ of the tumor (%ID T_Max_) was compared to the%ID_Max_ –BG (%ID BG_Max_) to better assess the injected dose activity. Raw values are shown in Table S3. The%ID_Max_ –liver (%ID L_Max_) and%ID_Mean_ –liver (%ID L_Mean_ served as a reference for high ^18^F-[FDG] uptake. Statistical analysis: Two-Way ANOVA post-hoc Tukey, * *p* < 0.05; ** *p*< 0.01; *** *p* < 0.001.

Hybrid imaging of positron emission tomography with 18F-fluorodeoxyglucose and computed tomography (^18^F-[FDG] PET/CT) is a key methodology to evaluate cancer patients [75]. ^18^F-[FDG] uptake reflects tumor physiology, tumor cell density and the distinction between active lesions or necrotic tissue [43]. In this work we performed the quantification of metabolic tumor volume (MTV), total lesion glycolysis (TLG) and the percentage of maximum (%ID_Max_) and average (%ID_Mean_) ^18^F-[FDG] injected dose activity in the tumor area. The tissue adjacent to the tumor (BG) and the liver were also evaluated to serve as reference and data normalization (Figure 5). The %ID of the record is an important reference to estimate the intensity of local metabolic activity, and the liver is one of the organs with the most constant ^18^F-[FDG] uptake [76].

MTV measures the volume of structures (e.g., tumors) that capture ^18^F-[FDG]. TLG can be defined as the sum of the MTV product of each lesion per its standardized uptake value, being an important parameter for the oncological prognosis. Normally, the higher the TLG the worse the individual’s prognosis, which can be correlated with a decrease in the overall survival rate and advancement of cancer staging [43,77,78].

According to the TLG values in Table 4, the best prognosis was observed in animals from the group treated with NLC_DTX_ IT + HGel-LDC TP which showed the lower metabolic activity of tumor cells in the neoplastic region (0.011). It is also noteworthy that the hybrid system (HGel-NLC_DTX_-LDC TP) group had a prognosis (TLG = 0.095) similar to that of the conventional treatment with DTX_T-HYD_ IT (0.105). A relatively low (0.288) metabolic activity of tumor cells was determined in the group treated with HGel-LDC TP-confirming the antitumor effect of lidocaine- but not in the animals from the HGel-NLC_DTX_ TP (1.287) group. As expected, the worst survival prognosis was observed in animals from the positive control group (1.558).

An additional advantage of micro-PET is the ability to assess the internal metabolic features of a tumor, which cannot be assessed by external calibrator measurements [78]. As shown in Table 4, even the tumor volume being bigger in the positive control, its MTV and TLG values were similar to those of treated groups such as the HGel-NLC_DTX_ TP. Accordingly to Tseng and co-workers [78] tumors larger than 1.000 mm^3^ develop central photopenia consistent with tumor necrosis. This effect may explain why tumors with large volumes have decreased tumor metabolic activity, and consequent lower in%ID_MAX_ values (Figure 5B). Therefore, the interpretation of data from tumors with volumes > 1.000 mm^3^ (as in the positive control) should be performed with caution, as tumor necrosis can affect the measurements of tumor volume and mean radiotracer accumulation. Photopenia also explains why larger tumors may have low MTV, as observed here for HGel-LDC TP (Table 4). These findings were confirmed by the presence of necrosis in the histopathological analysis (see Section 3.6).

Normally, undifferentiated tumor cells have more aggressive neoplastic characteristics and are more metabolically active than differentiated ones [78,79]. The relation between %ID^[T/BG]^_Max_ and %ID^[T/BG]^_Mean_ ratios (Table 4), can signal a transition from differentiated to undifferentiated tumor cells, when %ID^[T/BG]^_Max_ is greater than %ID^[T/BG]^_Mean_ [80]. From a clinical point of view this is very important, since undifferentiated cells generally do not respond well to chemotherapy being at a more advanced stage of the disease. [78]. As discussed above, photopenia, probably caused by tumor necrosis, may have interfered with the quantification of %ID values in the positive control and HGel-LDC *TP* groups, curbing the effective analyzes of cell differentiation in those groups. However, for the HGel-NLC_DTX_-LDC TP, DTX_T-HYD_ and NLC_DTX_ IT + HGel-LDC TP groups, Table 4 shows signs of high cell differentiation (%ID^[T/BG]^_Max_ < %ID^[T/BG]^_Mean_*)* in addition to *low* TLG values, as discussed before. An unexpected result was obtained with the HGel-NLC_DTX_ TP group that showed the highest %ID^[T/BG]^_Max_/%ID^[T/BG]^_Mean_ ratio (5.205/4.752) suggesting the occurrence of transitions from differentiated cells to undifferentiated cells and neoplasm evolution. 

Overall, the micro-PET/CT results confirm macroscopic observation (tumor volume), revealing a prognosis profile of: NLC_DTX_ IT + HGel-LDC TP > DTX_T-HYD_ ≥ HGel-NLC_DTX_-LDC TP > HGel-LDC TP ≥ HGel-NLC_DTX_ TP. Since the best results were achieved in the NLC_DTX_ IT + HGel-LDC TP group, we hypothesize that the low efficacy observed with HGel-NLC_DTX_ TP indicates that the release of docetaxel from the hydrophilic xanthan-chitosan hydrogel is a limiting step—unlike what was observed for lidocaine (HGel-LDC TP), a more polar compound. 

### 3.5. Screening of Treatments’ Adverse Effects 

#### 3.5.1. Biochemical Analyses

The biochemical analyses of alanine transaminase (ALT), aspartate aminotransferase (AST), creatinine and urea were performed in the blood of treated groups of animals (Table 5). Pathological processes in the liver can increase the activity of serum ALT and AST, caused by infections or chemical agents, such as antineoplastic drugs [79] while changes in serum levels of creatinine and urea are indicative of renal overload, nephrotoxicity and acute renal failure [80,81]. Overall, from a clinical point of view the levels of ALT, AST, urea, or creatinine remained normal after all treatments. 

**Figure 6:**
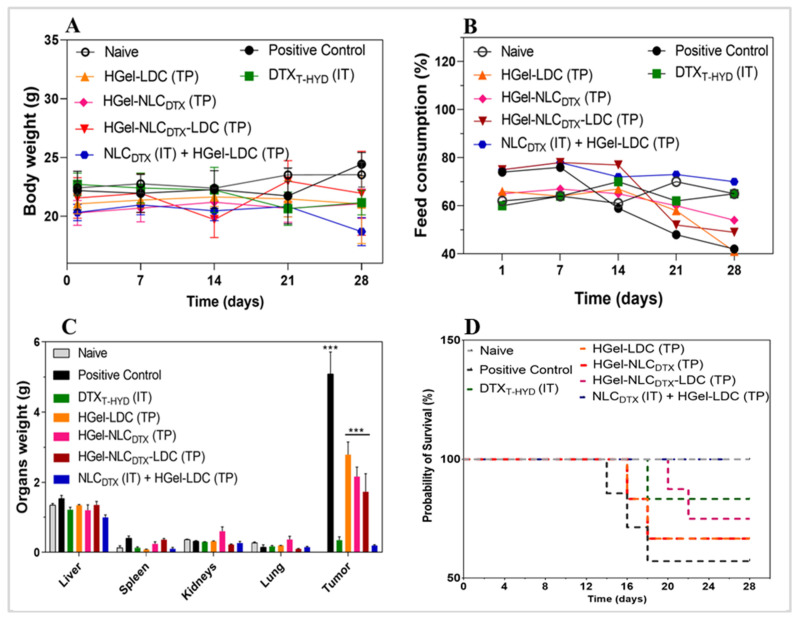
Evaluation of treatments and their adverse effects over C57BL/6J mice with melanoma. (**A**) Variation of body weight (**B**) Analysis of feed intake. (**C**) Weight of the organs (liver, spleen, kidneys, lung) and tumor region. (**D**) Kaplan–Meier survival curves for the different treated groups: naive, positive control (PBS group), positive control, DTX_T-HYD_ IT and nanohybrid hydrogel groups HGel-LDC TP, HGel-NLC_DTX_ TP, HGel-NLC_DTX_-LDC TP and NLC_DTX_ IT + HGel-LDC TP. Statistical analysis: Two-Way ANOVA post-hoc Bonferroni, *** *p* < 0.001; *n* = 5.

Additionally, mice weight and feed consumption were not affected by the treatments (as discussed further in Figure 6) showing the negligible systemic toxicity of these different treatments, in the administered doses.

#### 3.5.2. Macroscopic Parameters

After 10 days of treatment, animals were euthanized, and the organs (liver, spleen, kidneys, lung and tumor area) were removed and weighed to assess possible anatomopathological macroscopic changes [83]. As shown in Figure 6C, no significant changes (*p* < 0.05) in the mass of liver, spleen, lung and kidney of the animals were observed, in comparison to the negative (healthy) control group. This means that no macroscopic evidence of hyperplasia, hepatomegaly and splenomegaly were detected. Also, the quantified mass of the tumor region was the highest in the animals from the positive control group and regressed in HGel-LDC TP, HGel-NLC_DTX_ TP and HGel-NLC_DTX_-LDC TP groups, as shown in Figure 6C. The mass of the tumor region treated with the commercial formulation (DTX_T-HYD_ IT) and NLC_DTX_ IT + HGel-LDC TP treatments were equivalent and significantly lower (*p* < 0.05) than that observed in other groups. These results corroborate those of the quantified tumor volume (Figure 4A, Table 4).

The survival rates of the animals were assessed by checking the time elapsed between the tumor induction and the exact time of the animal’s death. In Figure 6D, it is possible to compare the cumulative animals’ survival as a function of the duration of the experiment (28 days total). The positive control (tumor) group showed the worst survival rate (40% or 2/5 alive animals). At the end of the test, survival rates of 60% (3/5 alive animals) were registered in HGel-LDC TP, HGel-NLC_DTX_ TP and HGel-NLC_DTX_-LDC TP groups, of 80% (4/5 alive animals) in DTX_T-HYD_ IT and of 100% in the treatment with NLC_DTX_ IT + HGel-LDC TP. These values are in good agreement with the micro-PET/CT data (Table 4), i.e., groups with lower TLG values (better prognosis) also had the higher survival rates. 

### 3.6. Histopathology

**Figure 7:**
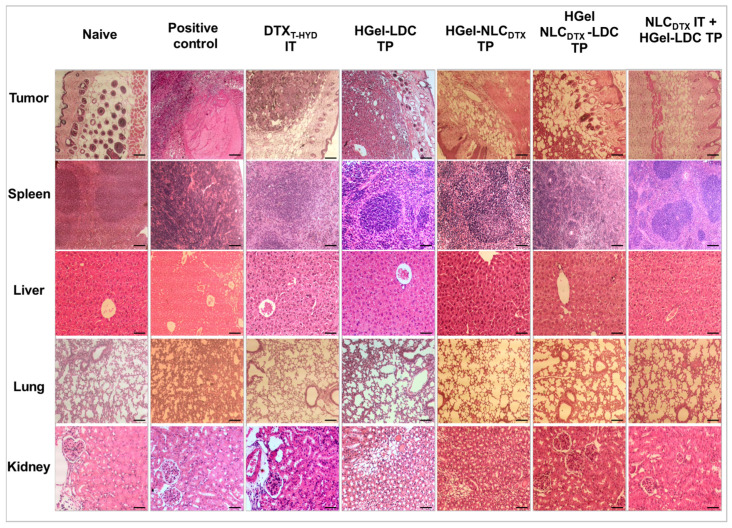
Histopathology sections of female C57BL/6J mice with orthotopically induced cancer (B16-F10 murine melanoma cells (except the naive group) and treated with: DTX_T-HYD_, HGel-LDC TP, HGel-NLC_DTX_ TP, HGel-NLC_DTX_-LDC TP and NLC_DTX_ IT + Gel-LDC TP. Analysis of the tumor region, spleen, liver, lung, and kidney; H & E, Scale bar = 50 µm, magnification: 100× or 400×).

The murine B16 melanoma model is the most commonly metastatic melanoma model used in preclinical studies [84]. B16-F10 cell line was generated as the tenth serial passage subclone of the B16 parent tumor line in C57BL/6J mice. In general, the in vivo assays employing intradermal/subcutaneous implants of B16-F10 cells in C57BL/6J mice result in aggressively growing tumors [35]. Histopathological analysis of the tumor region and organs—spleen, liver, lung, kidney—of the animals treated with either DTX_T-HYD_, HGel-LDC TP, HGel-NLC_DTX_ TP, HGel-NLC_DTX_-LDC TP and NLC_DTX_ IT + HGel-LDC TP was performed, as shown in Figure 7.

The degree of tumor tissue invasion was defined according to the Clark level scores, where: I: cancer is only in the epidermis; II: invasion to the papillary dermis; III: tumor fills the entire papillary dermis, without invading the reticular dermis; IV: invasion of the reticular dermis and V: invasion of the hypodermis [85]. The histological parameters that considered the tumor response to treatment were classified as: necrosis [86], reduction in the size of the neoplastic region [87], inflammatory infiltrate [88] and stromal fibroplasia [89]. 

It is known that the responses to therapy can be partial—when only part of the tumor regressed, or complete—when no more traces of the tumor are detected. Tumors that grow too fast can suffer spontaneous necrosis due to the hypoxia and nutrient (e.g., glucose) deficiency caused by scarce blood supplies [90]. This necrosis usually occurs at the center of the tumor, but in general it is not possible to distinguish between spontaneous and therapy-induced necrosis [90]. However, a therapy-induced necrosis is generally higher and more diffuse than spontaneous necrosis [88,91], as observed herein.

- Tumor

The Naive group didn’t exhibit any tumor, so its image refers to normal skin, with well-defined epidermis, dermis, and hypodermis (left upper image of Figure 7). The melanocytes had evident cytoplasm, with some melanin located in the space between the keratinocytes of the basal layer and the epidermis, of usual histological aspects. 

The positive control group had the worst prognosis, being classified as level V from the Clark score scale. In this group, the tissue region was thick, ulcerated and necrotic, with structural destruction of the epidermis. Blood vessels, typical of the angiogenesis were also observed. Histopathological findings show non equidistant melanocytic cells in the epidermis with different shapes and sizes (increased cytoplasm) and intense melanin production.

In the DTX_T-HYD_ group both levels II and III Clark scores were present. Histopathological analysis revealed diffuse necrosis on tumor site, with punctual foci of non-equidistant melanocytic cells, in addition to stromal fibroplasia with moderate inflammatory infiltrate, that could be a sign of the effectiveness of the therapy. 

The images from the NLC_DTX_ IT + HGel-LDC TP group were classified as levels I and II in the Clark scores scale. This treatment provided the best histological prognosis. Histopathological analysis showed a punctual and limited focus of neoplastic melanocytic cells with intense (peri and intratumor) lymphocytic infiltration. The regions exhibited stromal fibroplasia and diffuse necrosis and apparently little affected epidermal surface. 

The slices from the HGel-LDC TP, HGel-NLC_DTX_ TP and HGel-NLC_DTX_-LDC TP groups were classified at Clark’s III and IV levels. These groups had diffuse tissue necrosis with foci of melanocytic cells and high melanin production. Histopathology revealed stromal fibroplasia with moderate inflammatory infiltrate, consisting of evident lymphocytes and melanophages (Figure 7).

- Organs (spleen, liver, lung and kidneys)

The Naive, HGel-NLC_DTX_-LDC TP e NLC_DTX_ IT + HGel-LDC TP groups showed normal spleen histological structures, with the presence of splenic trabeculae, white and red pulps with usual aspects. The positive control group showed hyperplasia, splenic white pulp with proliferation of spindle cells of multinucleate aspects, intense inflammatory infiltrate, and metastasis. In the animals treated with DTX_T-HYD_, HGel-LDC TP and HGel-NLC_DTX_ TP, intense inflammatory infiltrate was observed (Figure 7).

Histological analyses of the liver revealed normal structures for the HGel-NLC_DTX_ TP, HGel-NLC_DTX_-LDC TP and NLC_DTX_ IT + HGel-LDC TP batches. The findings revealed the presence of well-defined epithelial cells (hepatocytes), nucleated cells close to the sinusoidal cells (probably Kupffer cells or macrophages) with normal characteristics. The images from the positive control group exhibited mild neutrophilic periportal hepatitis, without necrosis and the presence of metastases. The slices from DTX_T-HYD_ IT and HGel-LDC TP groups had evident inflammatory infiltrates (Figure 7). These analyses corroborate the observed changes in the hepatic markers ALT and AST (Table 5) for these groups.

In the histological evaluation of the lung, the naive group showed no abnormalities, with well-defined alveolar sacs, bronchi and bronchioles, usual macrophage or pneumocyte cells. The positive control group slices revealed moderate inflammatory infiltrate and pneumocyte hyperplasia, pulmonary edema with accumulation of protein fluid in the alveolar spaces and foci of pulmonary metastases. In the samples from the DTX_T-HYD_, HGel-LDC TP, HGel-NLC_DTX_ TP, HGel-NLC_DTX_-LDC TP e NLC_DTX_ IT + Gel-LDC TP groups mild inflammatory infiltrate and pneumocyte hyperplasia were detected.

In all the evaluated treatments, the kidneys showed usual histological aspects with medullary and cortical regions containing peritubular capillaries, renal corpuscles, plus distal and proximal contorted tubules with well-defined cubic epithelium.

In summary, histopathological findings showed that the positive control group had hepatic, splenic and pulmonary lesions, with distal metastases in the lung, spleen and liver, plus an ulcerative and necrotizing primary tumor. The B16-F10 cell line has shown similar metastatic behavior to that of human melanoma, where metastases in bones, lungs, liver and spleen are observed [92]. 

For all the treated groups (DTX_T-HYD,_ HGel-LDC TP, HGel-NLC_DTX_ TP, HGel-NLC_DTX_-LDC TP and NLC_DTX_ IT + HGel-LDC TP) the presence of diffuse necrosis in the primary tumor, peri and intratumor inflammatory infiltrates and stromal fibroplasia suggest therapeutic efficacy. Furthermore, in these treatments, there was no evident melanocytic metastasis in the analyzed sections. None of the treatments resulted in complete eradication of the tumor, since neoplastic cells were observed in all groups. However, the therapy with DTX_T-HYD_ and NLC_DTX_ IT + HGel-LDC TP had the smallest tumor area, with the best prognosis.

## 4. Conclusions

A hybrid hydrogel (HGel- NLC_DTX_) with anesthetic and antineoplastic effects was developed. It was composed of docetaxel (0.5%) loaded in nanostructured lipid carriers (NLC) plus a xanthan-chitosan polymeric matrix containing lidocaine (2%). CryoEM analyses of hybrid hydrogel revealed preservation of the nanoparticles structure even after insertion into the biopolymer matrix. Such system also exhibited pseudoplastic properties, as desired for stable hydrogels. Cell viability tests showed that the cytotoxicity of free DTX was reduced after encapsulation in the hybrid formulation, as a result of sustained drug release. In vivo assays indicated that the hybrid hydrogel was able to inhibit the tumor growth in an equivalent manner to the conventional (free DTX) treatment. Moreover, the treatment with the hybrid hydrogel showed no adverse effects, as revealed by physical, biochemical and histopathological parameters. These results validate the proposal that docetaxel loaded by NLC associated with lidocaine-in-hydrogel can be an alternative and promising biocompatible formulation for the treatment of melanoma. The results also revealed interesting lidocaine (antitumor and analgesic) effects for the melanoma therapy.

## Figures and Tables

**Table 1:** Characterization of NLC formulations with (NLC_DTX_) and without DTX (NLC_CTRL_) in terms of size, polydispersity index (PDI), zeta potential (ZP), encapsulation efficiency (%EE) and drug loading (%).

Formulation	Size (nm)	PDI	ZP (mV)	%EE	Drug Loading (%)
NLC_CTRL_	222.6 ± 8.5	0.15 ± 0.04	−25.9 ± 0.2	-	-
NLC_DTX_	214.0 ± 10.9	0.09 ± 0.01	−24.2 ± 0.3	97.3 ± 2.6	7.47 ± 0.26

**Table 2:** IC_50_ values of docetaxel (DTX) and lidocaine (free or encapsulated) against NIH-3T3, HaCaT, B16-F10 and SK-MEL-103 cells, after 24 h of treatments, as measured through the MTT assay. Analysis made with the GraphPad Prisma 6.04 software (mean ± SD, *n* = 3) of the values taken from the curves in Figure 2.

	Non-Cancerous Cell Lines				Cancer Cell Lines			
	NIH-3T3 (Murine)		HaCaT (Human)		B16-F10 (Murine)		SK-MEL-103 (Human)	
Formulations	IC_50_ of DTX (µmol L^−1^)	IC_50_ of LDC (mmol L^−1^)	IC_50_ of DTX (µmol L^−1^)	IC_50_ of LDC (mmol L^−1^)	IC_50_ of DTX (µmol L^−1^)	IC_50_ of LDC (mmol L^−1^)	IC_50_ of DTX (µmol L^−1^)	IC_50_ of LDC (mmol L^−1^)
DTX_T-HYD_	0.32 ± 0.53	-	0.03 ± 0.03	-	0.40 ± 0.48	-	0.33 ± 0.45	-
LDC	-	4.95 ± 5.60	-	2.67 ± 2.92	-	6.13 ± 5.44	-	3.26 ± 1.84
NLC_DTX_	2.71 ± 4.53	-	2.29 ± 2.84	-	3.53 ± 4.27	-	5.27 ± 3.47	-
NLC_DTX_ + LDC	2.63 ± 2.95	3.29 ± 3.69	1.83 ± 3.92	2.83 ± 3.53	2.08 ± 1.76	1.30 ± 2.21	1.32 ± 2.25	2.18 ± 3.21
HGel-NLC_DTX_	1.24 ± 2.70	-	3.72 ± 3.09	-	2.15 ± 1.54	-	1.55 ± 1.00	-
HGel-NLC_DTX_-LDC	0.71 ± 1.43	0.86 ± 1.73	0.29 ± 0.53	0.34 ± 0.68	0.81 ± 1.34	0.97 ± 1.65	0.28 ± 0.23	0.32 ± 0.10

**Table 3:** Tail-flick test in mice. Percent Maximum Possible Effect (MPE), Area under the curve (AUC) and time of anesthesia for the hydrogel lidocaine formulations (HGel-LDC), hydrogel plus NLC_CTRL_ (HGel-NLC_CTRL_) and hydrogel plus NLC and docetaxel (HGel- NLC_DTX_-LDC).

Formulation	MPE ± SD (%)	AUC ± SD	Time of Anesthesia (min)
HGel-LDC	88.48 ± 11.39	3545.8 ± 821.1	150
HGel-NLC_CTRL_-LDC	88.85 ± 15.84	3882.8 ± 805.2	150
HGel-NLC_DTX_-LDC	88.06 ± 11.65	4377.8 ± 950.8	150

Statistical analysis (Tukey’s multiple comparisons test, *n* = 5) showed no significant differences among the groups (HGel-LDC vs. HGel-NLC_CTRL_-LDC vs. HGel-NLC_DTX_-LDC).

**Table 4:** Values estimated by micro-PET/CT analysis of the percent ratio of the maximum or mean injected dose between the tumor region and the adjacent tissue (%ID^[T/BG]^_Max_ and %ID^[T/BG]^_Mean_), or liver (%ID^[T/L]^_Max_ and %ID^[T/L]^_Mean_); tumor metabolic volume (MTV) and total lesion glycolysis (TLG). The anatomical volume of the tumor (TV) measured with an external caliper is also given.

	Micro-PET/CT						Caliper
Formulations	%ID^[T/BG]^_Max_	%ID^[T/BG]^_Mean_	%ID^[T/L]^_Max_	%ID^[T/L]^_Mean_	MTV	TLG	TV (mm³)
Positive Control	9.169	11.336	2.812	2.570	0.633	1.558	3741.68
DTX_T-HYD_ IT	2.012	3.354	1.457	1.795	0.051	0.105	41.87
HGel-LDC TP	8.350	8.512	2.498	2.209	0.199	0.288	752.33
HGel-NLC_DTX_ TP	5.205	4.752	1.060	0.731	0.728	1.287	544.01
HGel-NLC_DTX_-LDC TP	3.641	4.129	0.518	0.390	0.034	0.095	155.23
NLC_DTX_ IT + HGel-LDC TP	1.230	1.806	0.410	0.423	0.012	0.011	42.60

Notations: T: tumor; BG: normal adjacent tissue (background); L: liver. %ID^[T/BG]^_Max_ = $\frac{\%\mathrm{ID}\;\mathrm{TMa}x\;}{\%ID\;\mathrm{BGMax}}$; %ID^[T/BG]^_Mean_ = $\frac{\%\mathrm{ID}\;\mathrm{TMean}\;}{\%\mathrm{ID}\;\mathrm{BGMean}}$; %ID^[T/L]^_Max_ = $\frac{\%\mathrm{ID}\;\mathrm{TMax}\;}{\%\mathrm{ID}\;\mathrm{LMax}}$; %ID^[T/L]^_Mean_ = $\frac{\%\mathrm{ID}\;\mathrm{TMean}\;}{\%\mathrm{ID}\;\mathrm{LMean}}$. The raw values of%IDMax and%IDMean in tumor, adjacent tissue and liver are shown in Table S3.

**Table 5:** Biochemical parameters in the serum of female C57BL/6J mice, after treatment with PBS (positive control), free docetaxel (DTX_T-HYD_) and hydrogel formulations (HGel-LDC, HGel-NLC_DTX_ TP, HGel-NLC_DTX_-LDC TP and NLC_DTX_ IT + HGel-LDC TP).

Groups	ALT (UI/L) (Rf:44.0–87.0)	AST (UI/L) (Rf:55.0–251.0)	Creatinine (mg/dL) (Rf: 0.48–1.10)	Urea (mg/dL) (Rf: 18.0–31.0)
Naive	52.0 ± 6.2	144.0 ± 10.5	0.66 ± 0.26	48.0 ± 18.0
Positive Control	171.0 ± 8.4	20.0 ± 5.7	0.68 ± 0.12	57.0 ± 14.1
DTX_T-HYD_ IT	28.0 ± 11.0	144.0 ± 3.4	0.15 ± 0.09	66.0 ± 17.9
HGel-LDC TP	135.0 ± 16.0	36.0 ± 9.0	0.57 ± 0.10	52.0 ± 16.3
HGel-NLC_DTX_ TP	34.0 ± 12.0	186.0 ± 6.2	0.57 ± 0.21	43.0 ± 15.5
HGel-NLC_DTX_-LDC TP	98.0 ± 14.4	213.0 ± 11.4	0.38 ± 0.14	52.0 ± 15.0
NLC_DTX_IT + HGel-LDC TP	45.0 ± 17.0	51.0 ± 8.4	0.52 ± 0.13	45.0 ± 19.0

NOTE: ALT, alanine aminotransferase. AST, aspartate aminotransferase. Values represented as mean ± standard error. Rf = reference range adapted from [82], Comp. Med. 2004.

## Data Availability

The data presented in this study are available on request from the corresponding author.

## Supplementary Materials

The following are available online at https://www.mdpi.com/article/10.3390/pharmaceutics13101552/s1, Table S1: Screening of the best concentrations of xanthan and chitosan biopolymers to be used as hydrogel excipients; Table S2. Rheological parameters (G’, G”, G’/G” and ƞ) for the prepared hydrogel formulations, measured at 32.5 °C; Table S3. Values estimated by micro-PET/CT analysis of the percent ratio of the maximum and mean injected dose in tumor (%ID TMax or%ID TMean), adjacent tissue (%ID BGMax or%ID BGMean) and liver (%ID LMax or%ID LMean); Figure S1. Colloidal stability of formulations with DTX (NLC_DTX_) and without (NLCCTRL) during 12 months of storage at ambient temperature. (A) Size (nm) and PDI; B) Zeta potential (ZP); Figure S2. Digital photos of the hydrogels prepared with different chitosan:xanthan ratios (see Table S1). (A) control hydrogels, without NLC; (B) An example of LDC incorporated in chitosan:xanthan hydrogel; (C,D) Whitish hydrogels, after NLC incorporation. (D) Sample 2 (see text) showing the consistency of the prepared hybrid (NLC-in-hydrogel) formulation; Figure S3. CryoEM micrographs of nanostructured lipid carriers without (NLCCTRL) and with docetaxel (NLC_DTX_). 100.000x, 120 kV; Figure S4. Viability of NIH-3T3 (A), HaCaT (B), B16-F10 (C) and SK-MEL-103 (D) cells after 72 h of treatment with DTXT-HYD, LDC, NLC_DTX_, NLC_DTX_ + LDC, HGel-NLC_DTX_ or HGel-NLC_DTX_-LDC at 0.02, 0.1, 0.5, 2 and 8 µmol mL−1 (equivalent DTX concentrations) and 0.03, 0.1, 0.6, 2.5 and 10 mmol L−1(equivalent LDC concentrations, evaluated by MTT assay. Data expressed as mean  ±  standard error. Two-Way ANOVA post-hoc Bonferroni (*** *p* < 0.001—* Treatment compared to control).; Figure S5. Tail-flick (analgesia) test. Maximum possible effect (MPE) as a function of time after treatment with hydrogels containing: 2% lidocaine (HGel-LDC), control NLC—without docetaxel—plus 2% lidocaine (HGel-NLCCTRL-LDC) or docetaxel in NLC plus 2% lidocaine (HGel-NLC_DTX_-LDC). For each formulation MPE significantly changed (*** *p* < 0.001) during the time course of the experiment (from 30 to 120 min), but no significant differences were registered among the formulations at any experimental time analyzed; Figure S6. Digital images of C57BL/6J mice with melanoma after treatment with the hybrid formulation.
