# Supplementary material for: Docetaxel and Lidocaine Co-Loaded (NLC-in-Hydrogel) Hybrid System Designed for the Treatment of Melanoma
Source: Pharmaceutics. 2021 Sep 24;13(10):1552. doi: 10.3390/pharmaceutics13101552 (PMC8537790; doi:10.3390/pharmaceutics13101552)
Supplement: Supplementary file 1 [file pharmaceutics-13-01552-s001.zip › pharmaceutics-1376707-supplementary.pdf]

pharmaceutics

MDPI

Supplementary Materials: Docetaxel and Lidocaine Co-Loaded (NLC-in-Hydrogel) Hybrid System Designed for the Treatment of Melanoma

Ludmilla David de Moura, Lígia N. M. Ribeiro, Fabíola V. de Carvalho, Gustavo H. Rodrigues da Silva, Priscila C. Lima Fernandes, Sérgio Q. Brunetto, Celso D. Ramos, Lício A. Velloso, Daniele R. de Araújo and Eneida de Paula

Table S1. Screening of the best concentrations of xanthan and chitosan biopolymers to be used as hydrogel excipients.

			(%, w:w)		
**Biopolymers**	**Sample 1**	**Sample 2**	**Sample 3**	**Sample 4**	**Sample 5**
Chitosan	1.5	1.0	0.8	0.5	0.3
Xanthan	2.5	3.3	3.6	4.1	4.5

Table S2. Rheological parameters (G′, G″, G′/G″ and η) for the prepared hydrogel formulations, measured at 32.5 °C.

	**G' (Pa)**	**G" (Pa)**	**G'/G″**	**η (mPa.s)**
**HGel$_{CTRL}$**	421.7	71.7	5.8	68090
**HGel-LDC**	450.3	81.3	5.5	72780
**HGel-NLC$_{CTRL}$**	114.1	25.5	4.5	18610
**HGel-NLC$_{CTRL}$-LDC**	126.6	32.1	3.9	20780
**HGel-NLC$_{DTX}$**	73.16	19.5	3.7	12050
**HGel-NLX$_{DTX}$-LDC**	86.53	21.5	4.0	14190

Table S3. Values estimated by micro-PET/CT analysis of the percent ratio of the maximum and mean injected dose in tumor (% ID T$_{Max}$ or % ID T$_{Mean}$), adjacent tissue (% ID BG$_{Max}$ or % ID BG$_{Mean}$) and liver (% ID L$_{Max}$ or % ID L$_{Mean}$).

			micro-PET/CT			
**Formulations**	**% ID T$_{Max}$**	**%ID T$_{Mean}$**	**%ID BG$_{Max}$**	**%ID BG$_{Mean}$**	**%ID L$_{Max}$**	**%ID L$_{Mean}$**
**Positive Control**	3.631	2.46	0.396	0.217	1.291	0.957
**DTX$_{T-HYD}$ IT**	2.342	2.043	1.164	0.609	1.607	1.138
**HGel-LDC TP**	2.121	1.447	0.254	0.17	0.849	0.655
**HGel-NLC$_{DTX}$ TP**	2.769	1.768	0.532	0.372	2.613	2.417
**HGel-NLC$_{DTX}$-LDC TP**	4.023	2.758	1.105	0.668	7.76	7.07
**NLC$_{DTX}$ IT + HGel-LDC TP**	0.948	0.894	0.771	0.495	2.31	2.111

* Notations: **T**, Tumor. **BG**, normal adjacent tissue. **L**, Liver.

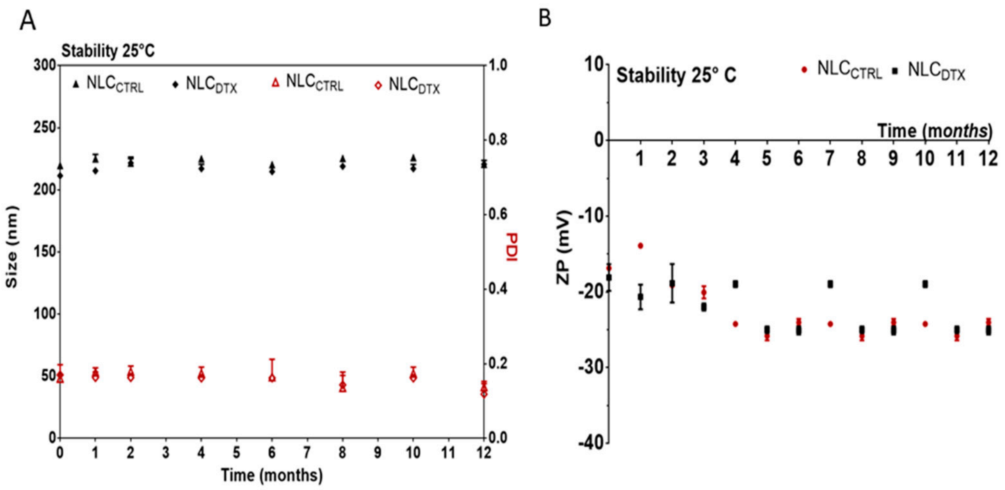

Figure S1. Colloidal stability of formulations with DTX (NLC$_{DTX}$) and without (NLC$_{CTRL}$) during 12 months of storage at ambient temperature. A) Size (nm) and PDI; B) Zeta potential (ZP).

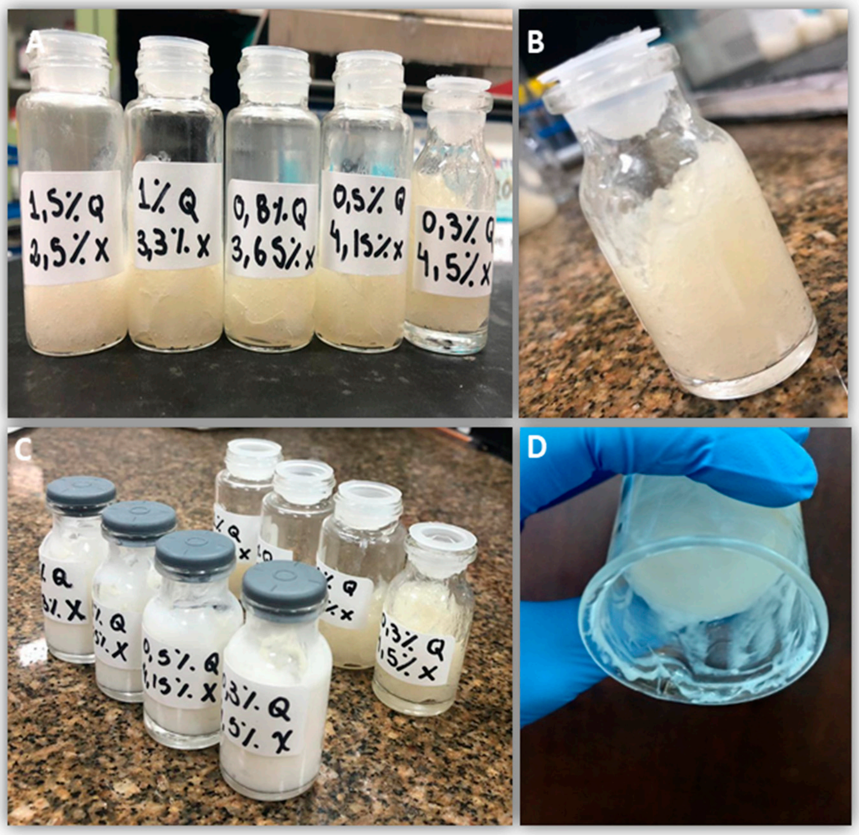

Figure S2. Digital photos of the hydrogels prepared with different chitosan:xanthan ratios (see Table S2). (A) control hydrogels, without NLC; B) An example of LDC incorporated in chitosan:xanthan hydrogel; (C, D) Whitish hydrogels, after NLC incorporation. D) Sample 2 (see text) showing the consistency of the prepared hybrid (NLC-in-hydrogel) formulation.

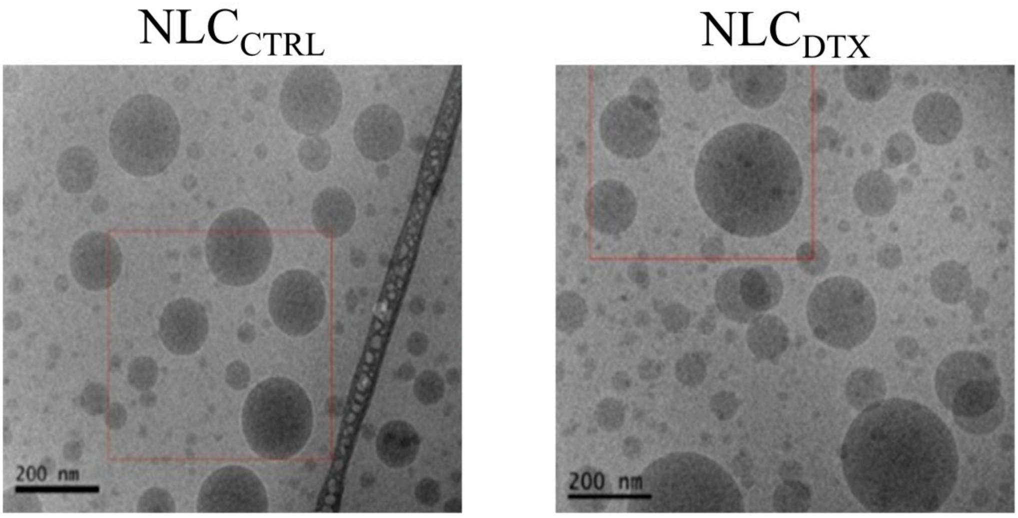

Figure S3. CryoEM micrographs of nanostructured lipid carriers without (NLC$_{CTRL}$) and with docetaxel (NLC$_{DTX}$). 100.000×, 120 kV.

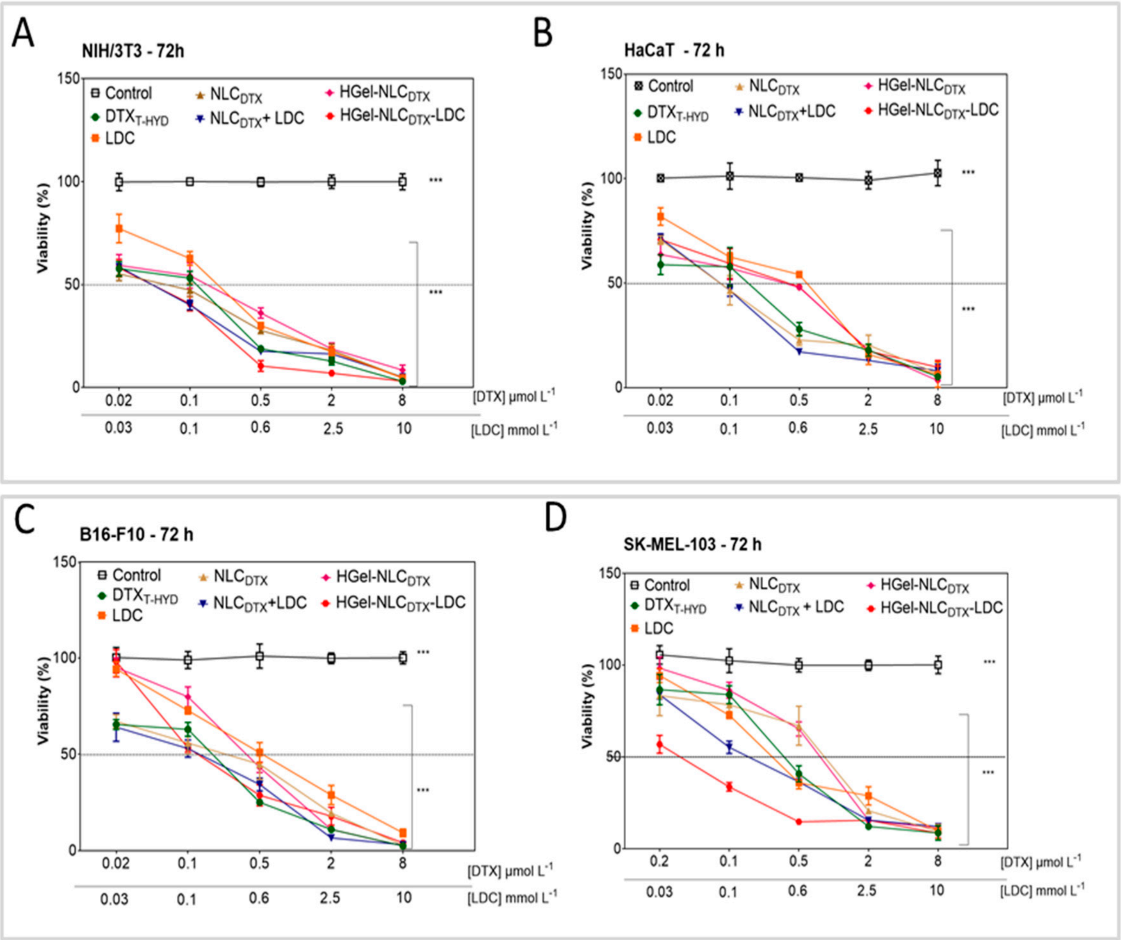

Figure S4. Viability of NIH-3T3 (A), HaCaT (B), B16-F10 (C) and SK-MEL-103 (D) cells after 72 h of treatment with DTX$_{T-HYD}$, LDC, NLC$_{DTX}$, NLC$_{DTX}$ + LDC, HGel-NLC$_{DTX}$ or HGel-NLC$_{DTX}$-LDC at 0.02, 0.1, 0.5, 2 and 8 µmol mL^{-1} (equivalent DTX concentrations) and 0.03, 0.1, 0.6, 2.5 and 10 mmol L^{-1}(equivalent LDC concentrations, evaluated by MTT assay. Data are expressed as mean ± standard error. Two-Way ANOVA post-hoc Bonferroni ($^{***}p < 0.001$ - *Treatment compared to control).

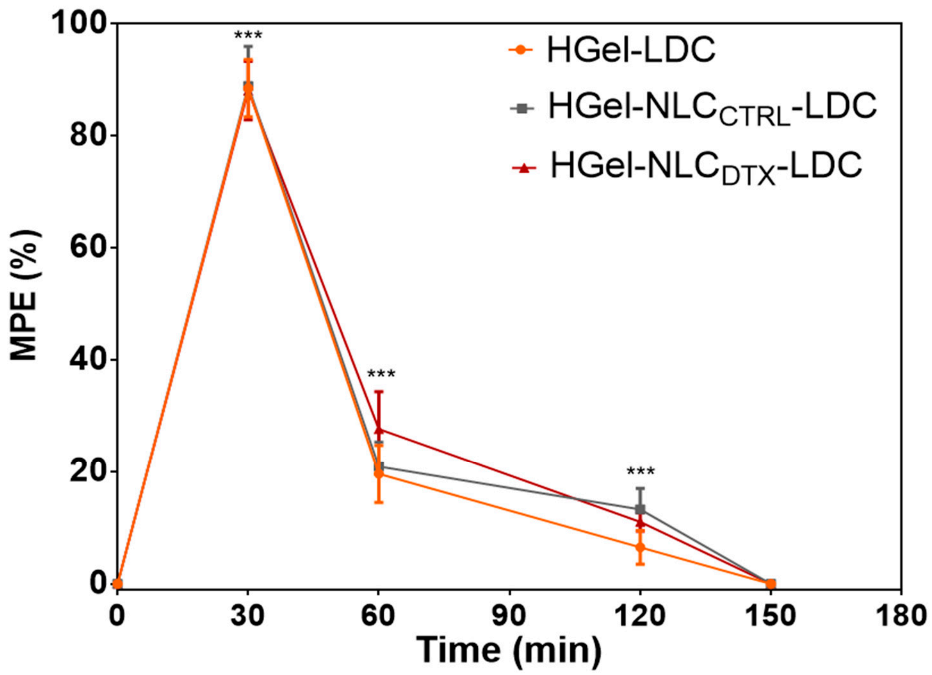

Figure S5. Tail-flick (analgesia) test. Maximum possible effect (MPE) as a function of time after treatment with hydrogels containing: 2% lidocaine (HGel-LDC), control NLC - without docetaxel - plus 2% lidocaine (HGel-NLC$_{CTRL}$-LDC) or docetaxel in NLC plus 2% lidocaine (HGel-NLC$_{DTX}$-LDC). For each formulation MPE significantly changed ($^{***}p < 0.001$) during the time course of the experiment (from 30 to 120 min), but no significant differences were registered among the formulations at any experimental time analyzed.

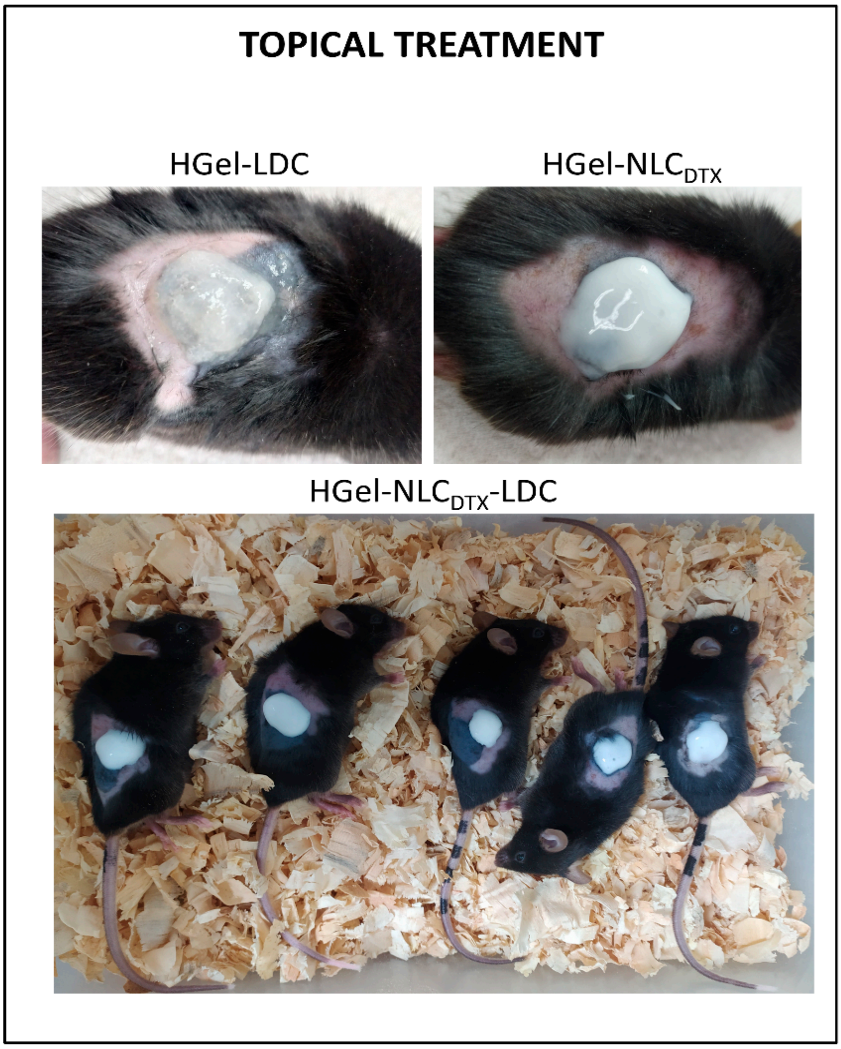

Figure S6. Digital images of C57BL/6J mice with melanoma after treatment with the hybrid formulation.
